# B6eGFPChAT mice overexpressing the vesicular acetylcholine transporter exhibit spontaneous hypoactivity and enhanced exploration in novel environments

**DOI:** 10.1002/brb3.139

**Published:** 2013-04-18

**Authors:** Paul M Nagy, Isabelle Aubert

**Affiliations:** 1 Brain Sciences, Biological Sciences, Sunnybrook Research Institute2075 Bayview Avenue, Toronto, Ontario, M4N 3M5, Canada; 2 Department of Laboratory Medicine and Pathobiology, University of Toronto1 King's College Circle, Toronto, Ontario, M5S 1A8, Canada

**Keywords:** Choline acetyltransferase, cholinergic, eGFP-ChAT, exploration, locomotion, VAChT

## Abstract

Cholinergic innervation is extensive throughout the central and peripheral nervous systems. Among its many roles, the neurotransmitter acetylcholine (ACh) contributes to the regulation of motor function, locomotion, and exploration. Cholinergic deficits and replacement strategies have been investigated in neurodegenerative disorders, particularly in cases of Alzheimer's disease (AD). Focus has been on blocking acetylcholinesterase (AChE) and enhancing ACh synthesis to improve cholinergic neurotransmission. As a first step in evaluating the physiological effects of enhanced cholinergic function through the upregulation of the vesicular acetylcholine transporter (VAChT), we used the hypercholinergic B6eGFPChAT congenic mouse model that has been shown to contain multiple VAChT gene copies. Analysis of biochemical and behavioral paradigms suggest that modest increases in VAChT expression can have a significant effect on spontaneous locomotion, reaction to novel stimuli, and the adaptation to novel environments. These observations support the potential of VAChT as a therapeutic target to enhance cholinergic tone, thereby decreasing spontaneous hyperactivity and increasing exploration in novel environments.

## Introduction

Cholinergic neurotransmission plays key roles in the central and peripheral nervous systems (Woolf and Butcher 2011). Cholinergic impairments in neurodegenerative diseases, especially in Alzheimer's disease (AD), have led to the development of several cholinergic-based therapeutic strategies. Aside from the well-characterized role of acetylcholine (ACh) in cognitive functions such as learning and memory, ACh availability has been shown to contribute to a number of physiological and behavioral functions including peripheral motor function (Ribeiro et al. 2006; Woolf and Butcher 2011) and locomotor activity (Di Chiara et al. 1994; Martins-Silva et al. 2011; Woolf and Butcher 2011). Specifically, clinical assessments and experimental models of AD revealed that decreased cholinergic tone can cause spontaneous hyperactivity including increased restlessness, coupled with increased anxiety in novel environments (Ognibene et al. 2005; Piccininni et al. 2005; McGuinness et al. 2010; Sterniczuk et al. 2010b; Bedrosian et al. 2011; Walker et al. 2011). Therefore, strategies to modify cholinergic tone may provide a means to regulate both spontaneous and novelty-induced locomotion (Mega et al. 1999).

Cholinergic neurotransmission is maintained through the appropriate synthesis, vesicular packaging, and release of ACh. Choline, sequestered through the high-affinity choline transporter (CHT), is transacetylated via the enzymatic activity of choline acetyltransferase (ChAT) and the precursor acetyl-coenzyme A (reviewed in Blusztajn and Wurtman 1983). Newly synthesized ACh is packaged into synaptic vesicles by vesicular acetylcholine transporter (VAChT) prior to its release to the synaptic cleft (Parsons 2000).

Genetic targeting has been used to create mouse models presenting deficiency in one or more cholinergic components, including VAChT (Prado et al. 2006; de Castro et al. 2009a; Guzman et al. 2011; Martins-Silva et al. 2011), ChAT (Misgeld et al. 2002; Brandon et al. 2004), CHT (Bazalakova et al. 2007), acetylcholinesterase (AChE) (Volpicelli-Daley et al. 2003) or through the modified expression of ACh receptors (Picciotto et al. 2000; Wess et al. 2007; Drenan et al. 2008, 2010). Until recently, most animal models of cholinergic enhancement have been limited to the pharmacological inhibition of ACh degradation in the synaptic cleft. The previously characterized B6eGFPChAT mouse model (Tallini et al. 2006; Nagy and Aubert 2012) allows for the evaluation of whether increasing the vesicular storage and release of ACh is sufficient to elicit changes in behavioral activity. B6eGFPChAT mice have four genomic copies of the cholinergic gene locus, which contains the VAChT and ChAT promoter and coding regions (Eiden 1998; Tallini et al. 2006; Nagy and Aubert 2012). In these mice, the transcription of transgenic ChAT is terminated and replaced by the enhanced green fluorescent protein (eGFP), while the transcription of the VAChT transgene remains operational. As such, VAChT is overexpressed, while levels of ChAT, CHT, and AChE are maintained, in cholinergic neurons (Nagy and Aubert 2012).

Here, the behavior of B6eGFPChAT mice was assessed in a panel of tests designed to elicit a variety of central and peripheral responses. We found that B6eGFPChAT mice have enhanced spontaneous activity and novelty-induced exploration. The results of this study support the notion that modulating VAChT levels modifies behavioral activity, highlighting the importance of ACh vesicular storage in the regulation of cholinergic neurotransmission and function.

## Materials and Methods

### Animals

For all studies, congenic male B6.Cg-Tg(RP23-268L19-EGFP)2Mik/J (B6eGFPChAT; Jackson Laboratories, Bar Harbour, ME) mice homozygous for the RP23-268L19-EGFP transgene were compared with sex and age-matched B6 controls. Separate cohorts of animals were used for the biochemical, immunohistological, and behavioral studies. For the behavioral panel, B6eGFPChAT (*N* = 11) and B6 (*N* = 9) mice were between 124 and 126 days of age at upon entry to this study, housed under identical conditions, and exposed to regular handling prior to and during the study. The behavioral panel was conducted sequentially in the following order: open field (Days 1–5), peripheral motor function (Day 9), Rotorod (Days 10–11), dark/light box (Day 18), and elevated plus maze (Day 48). A subset of this cohort (*N* = 8 per genotype) were used for calorimetry (Days 24–28; Days 37–41). Presence of the transgene was confirmed using conventional polymerase chain reaction (PCR) and primers as previously described (Tallini et al. 2006), and by the expression of eGFP observed during immunofluorescence microscopy protocols. All animal protocols were approved by the Animal Care Committees of Sunnybrook Research Institute and the University of Western Ontario, and experiments were performed according to the guidelines set by the Canadian Council on Animal Care and the Animals for Research Act of Ontario.

### Immunofluorescence microscopy

B6eGFPChAT mice were concurrently anesthetized with a mixture of ketamine (75 mg/kg) and xylazine (10 mg/kg). The mice were then perfused intracardially with saline, followed by fixation with 4% paraformaldehyde in 0.1 mol/L phosphate buffer. Brains were removed, postfixed overnight, and equilibrated in 30% sucrose. Coronal sections were cut at 30 μm and collected in 96-well plates filled with cryoprotectant solution (50 mmol/L phosphate buffer; 25% (v/v) glycerin; 30% (v/v) ethylene glycol; pH 7.4). Sections were blocked using 0.3% bovine serum albumin in phosphate-buffered saline and incubated with a primary antibody against VAChT (guinea pig polyclonal AB1588; 1:1000 dilution; Millipore, Temecula, CA) followed with a donkey anti-guinea pig Cy3 antibody to reveal VAChT immunoreactivity (1:500; Jackson ImmunoResearch Laboratories, Inc., West Grove, PA). Fluorescent labeling was detected by confocal microscopy (Zeiss Axiovert 100M, LSM 510; Carl Zeiss, Don Mills, Canada).

### Western blot

Proteins (25 μg total protein per lane) from B6eGFPChAT (*N* = 3) and B6 (*N* = 3) cortical, striatal and hippocampal brain homogenates were separated by 10% sodium dodecyl sulfate polyacrylamide gel electrophoresis and transferred to a nitrocellulose (Trans-Blot transfer medium; Bio-Rad Laboratories, Richmond, CA) or polyvinylidene fluoride membrane (Immobilon-P, Millipore). The membranes were then blocked with tris-buffered saline with Tween-20 (TBST) (20 mmol/L Tris HCl; 137 mmol/L NaCl; 0.1% (v/v) Tween 20; pH 7.6) containing 5% skim milk. The membranes were washed in TBST and incubated with guinea pig anti-VAChT (AB1588, Millipore), anti-ChAT (AP144P, Millipore), or anti-CHT (AB5966, Millipore) antibodies overnight at 4°C. Following successive washing with TBST, the membranes were incubated with the appropriate horseradish peroxidase-conjugated secondary antibody. Immunoreactive signals were detected using the SuperSignal West Dura enhanced chemiluminescence system (Pierce, Rockford, IL).

To quantify the relative amount of protein expression, blots were stripped and reprobed with antibodies against GAPDH (H86504M, Meridian Life Science, Memphis, TN) for 1 h followed by a horseradish peroxidase-conjugated secondary antibody for an additional hour. Signal intensities were analyzed using GeneTools software (Syngene, Frederick, MD) and normalized to GAPDH. The relative amount of VAChT, ChAT, and CHT protein in B6eGFPChAT tissue homogenates was expressed as a percent of protein present in B6 control tissue. Mean normalized densitometry values were analyzed by Student's *t*-test to compare genotypes.

### Spontaneous activity and indirect calorimetry

B6eGFPChAT (*N* = 8) and B6 (*N* = 8) mice were placed in comprehensive lab animal monitoring system (CLAMS) metabolic cages (Columbus Instruments, Columbus, OH). These metabolic chambers monitor activity and metabolic performance. Following entry into the cages, the mice were allowed to acclimatize to the environment for 14–17 h prior to data collection. High-resolution real time activity data along with metabolic measurements collected every 10 min were acquired during the 12 h light cycle (0700 and 1900 h) and 12 h dark cycle (1900 and 0700 h). The metabolic measurements included the volume of carbon dioxide produced (VCO_2_), the volume of oxygen consumed (VO_2_), the respiration exchange ratio (RER = VCO_2_/VO_2_), and the caloric (heat) value (([(3.815 + 1.232 × RER) × VO_2_] × 1000)/mouse weight). Sleep analysis was conducted using the Oxymax software (Columbus Instruments, Columbus, OH) as previously described and validated (Pack et al. 2007). The sleep threshold was set to 180 sec of ≤10 activity counts. The data are represented in ∼30 min intervals and analyzed using repeated measures two-way analysis of variance (ANOVA) or as the mean values over each 12 h period and analyzed using Student's *t*-test.

### Dark/light box

Each mouse was placed into an automated activity monitor (Accuscan Instruments, Inc., Columbus, OH) that was separated into an enclosed dark region (20 × 40 cm) and an open light region (20 × 40 cm). The two regions were separated by an opening (10 × 15 cm) where mice were placed facing the dark region and allowed to explore for 10 min between 2000 and 2200 h. Activity (converted from infrared beam breaks to cm) in each of the two regions along with transitions between the regions were measured over the trial duration. Mean distance values were analyzed by Student's *t*-test. The proportional time and distance spent in the light field and the number of transitions were analyzed by the Mann–Whitney *U* test.

### Novel environment locomotion

Locomotor activity was measured using an automated activity monitor (Accuscan Instruments, Inc., Columbus, OH). Experiments were performed between 1000 and 1600 h. Mice were allowed to explore the locomotor activity chamber (20 × 20 cm) for 2 h. Activity (converted from infrared beam breaks to cm) was measured at 5 min intervals. Measurements of activity were analyzed using repeated measures two-way ANOVA while cumulative means were assessed by the Student's *t*-test.

### Elevated plus maze

Anxiety-like and exploratory behavior were evaluated using an elevated plus maze 50 cm above the floor with four arms 30 cm long and 5 cm wide (two darkened and enclosed with 40 cm walls). Mice were placed into the center of the maze facing one of the open arms. The accumulated time and distance spent on the open and closed arms, along with the entries into each of the arms was recorded over a single trial of 5 min using the automated tracking system (AnyMaze, Stoelting, Wood Dale, IL). The percentage of time spent on each of the arms and the number of entries into the arms were analyzed using Student's *t*-test or Mann–Whitney *U* test as parameters measuring anxiety-like behavior.

### Rotarod

Mice were placed on a stationary rod of an automated rotarod apparatus (SD Instruments, San Diego, CA). The rotation of the rod was then initiated at the speed of 5 rpm, which accelerated at a rate of 10 rpm/min to 35 rpm over the course of 3 min. Latency to fall was automatically recorded using infrared beam break as the animal fell from the rod. Mice were tested on 10 trials during the first day, and four trials the next day, each with 15 min intertrial intervals. Results were analyzed by repeated measures ANOVA.

### Grip strength

Forelimb grip strength was measured using a horizontally mounted digital force gauge (Chatillon, Largo, FL). Mice held by the base of their tails were slowly lowered and allowed to grasp a triangular bar attached to the gauge. The mice were then pulled backwards along the horizontal plane of the gauge. The peak tension of 10 successive trials was collected. Mean peak tension results for each genotype were analyzed by Student's *t*-test.

### Hanging wire

Each mouse was placed on a wire cage top (square ½ inch mesh) which was gently shaken once to encourage the mice to grasp. The wire cage top was slowly inverted and suspended 40 cm above the base of a padded Plexiglas box. The mice were given three trials up to 300 sec with an intersession interval of 30 sec. The time it took each mouse to fall from the cage top was recorded. The mean trial hanging time results for each genotype were analyzed using repeated measures ANOVA and mean cumulative hang time over each of the trials were analyzed by Student's *t*-test.

## Results

### VAChT is overexpressed in the B6eGFPChAT mouse

**Figure 1:**
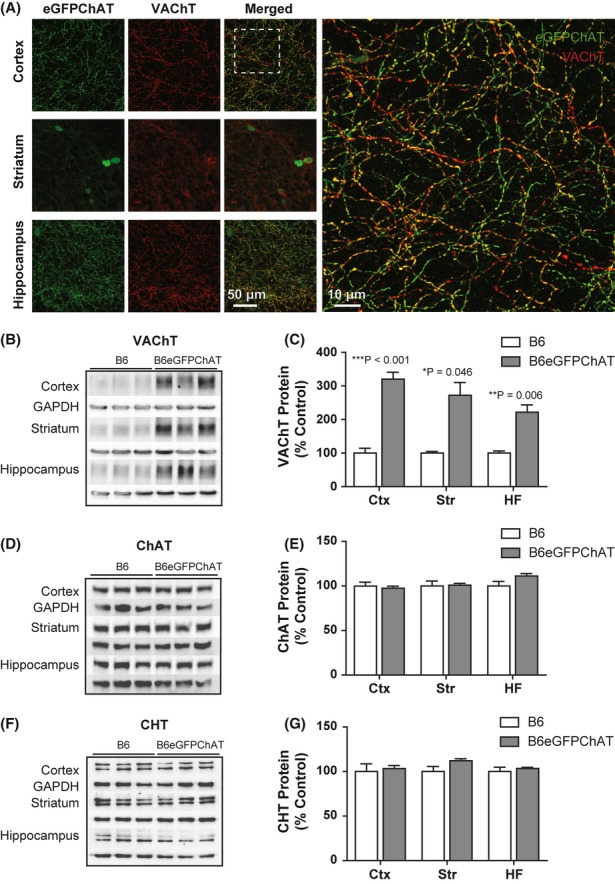
VAChT protein is overexpressed in B6eGFPChAT mice. (A) RP23-268L19-EGFP transgene is expressed throughout the central nervous system of B6eGFPChAT mice including cortical, striatal, and hippocampal regions (green). Punctate VAChT immunoreactivity is present in cholinergic cell bodies and processes (red). (B and C) Representative immunoblots for VAChT (B) and densitometry quantification (C) of immunoblot reveals significant two- to threefold overexpression of VAChT in the cortex, striatum, and hippocampus of B6eGFPChAT mice compared with B6 control mice. (D and E) Representative immunoblot for ChAT (D) and densitometry quantification (E) shows no significant difference in ChAT expression between genotypes in the analyzed regions. (F and G) Representative immunoblot for CHT (F) and densitometry quantification (G) shows no significant difference in CHT expression between genotypes in the analyzed regions. *P < 0.05; **P < 0.01; ***P < 0.005 compared with B6 control mice.

In the cortex, striatum, and hippocampus, VAChT staining presented as punctate fluorescence along ChAT positive fibers and in cell bodies of the striatum (Fig. 1A). Our previous observations in 3-month-old B6eGFPChAT mice (Nagy and Aubert 2012) revealed enhanced VAChT protein expression and here, we confirm that at 6 months of age, VAChT overexpression is sustained. The expression of VAChT in B6eGFPChAT mice was compared with B6 controls using Western blot analysis to detect cholinergic immunoreactivity in various regions of the central nervous system. Western blot targeting VAChT revealed a diffuse doublet at the predicted size of 70 kDa (Fig. 1B). Quantification of the VAChT band intensity revealed a significant two- to threefold increase of VAChT protein in B6eGFPChAT compared with B6 control mice (Fig. 1C). The enhanced level of VAChT protein was found in the cortex (*t*_(4)_ = 8.752; *P* = 0.001), striatum (*t*_(4)_ = 4.494; *P* = 0.046), and hippocampal formation (*t*_(4)_ = 5.323; *P* = 0.006) (Fig. 1D). Western blots and quantification of ChAT (Fig. 1D and E) and CHT (Fig. 1F and G) revealed no significant change in protein expression in any of the regions that were analyzed.

### B6eGFPChAT mice exhibit unaltered motor function and coordination

**Figure 2:**
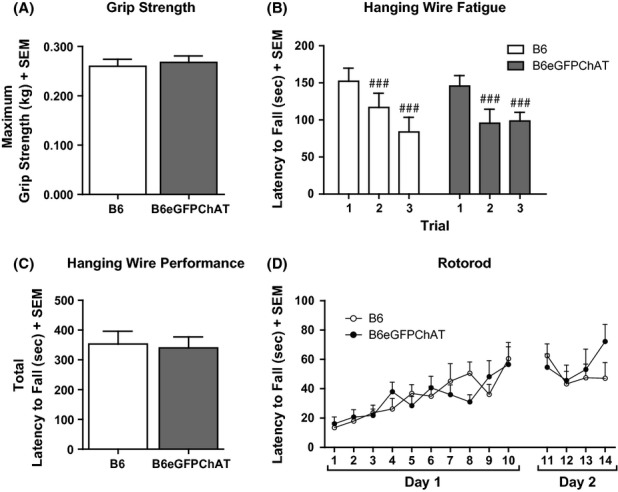
Neuromuscular function and coordination in B6eGFPChAT mice. (A) Maximum forelimb grip strength measured in B6eGFPChAT (*N* = 11) and B6 control mice (*N* = 9). (B and C) Time spent hanging upside-down from a wire grid in each of three consecutive trials (B) and cumulative time (C) for B6eGFPChAT (*N* = 11) and B6 control mice (*N* = 9). (D): Latency to fall from an accelerating rod (5–35 rpm; rate of 10 rpm/min) over 10 trials on day 1, followed by 4 trials on day 2. ^###^*P* < 0.005 compared with trial 1.

To measure the effect of increased VAChT on peripheral motor function, we first assessed forelimb grip strength using a digital tension gauge. B6eGFPChAT mice produced a peak tension of 0.268 kg which was not found to be significantly different from B6 control mice that produced 0.260 kg of peak tension (*t*_(18)_ = 0.416; *P* = 0.682) (Fig. 2A). In addition, no statistical difference was found between B6eGFPChAT and B6 control mice when measuring wire hang fatigue (two-way repeated measures ANOVA revealed no significant genotype factor, *F*_(1,36)_ = 0.052; *P* = 0.822, and the expected trial factor, *F*_(2,36)_ = 11.04; *P* < 0.001) (Fig. 2B) or total hanging time performance (*t*_(18)_ = 0.229; *P* = 0.822) (Fig. 2C).

We considered that the effect of VAChT overexpression might only be detectable during activities combining endurance, fine motor coordination, and balance. As such, performance on the rotarod was assessed through the latency to fall off the rotating cylinder. Both B6eGFPChAT and B6 control mice improved significantly from trial 1 to trial 10 (two-way repeated measures ANOVA trial factor, *F*_(9,162)_ = 8.653; *P* < 0.001), demonstrating that both strains significantly improved motor coordination over time (Fig. 2D). However, no significant effect of genotype (*F*_(1,162)_ = 0.013; *P* = 0.910) or interaction (*F*_(9,162)_ = 1.273; *P* = 0.256) was found. Motor skill retention was assessed using four additional trials for 24 h following the training sessions. In this paradigm, no significant differences were found between the latency to falling during Trial 10 (Day 1) and Trial 11 (Day 2) for B6eGFPChAT or B6 control mice (two-way repeated measures ANOVA *F*_(1,18)_ = 0.201; *P* = 0.659). Similarly, no genotype effect on performance was observed during the four trials performed during Day 2 (*F*_(1,54)_ = 0.366; *P* = 0.553) (Fig. 2D). Taken together, these data suggest that B6eGFPChAT mice have maintained motor function and learning compared with B6 control mice, and that elevated VAChT-mediated ACh vesicular packaging as observed in B6eGFPChAT mice is not sufficient to improve these normal motor functions.

### B6eGFPChAT mice display spontaneous hypoactivity in a home cage environment

**Figure 3:**
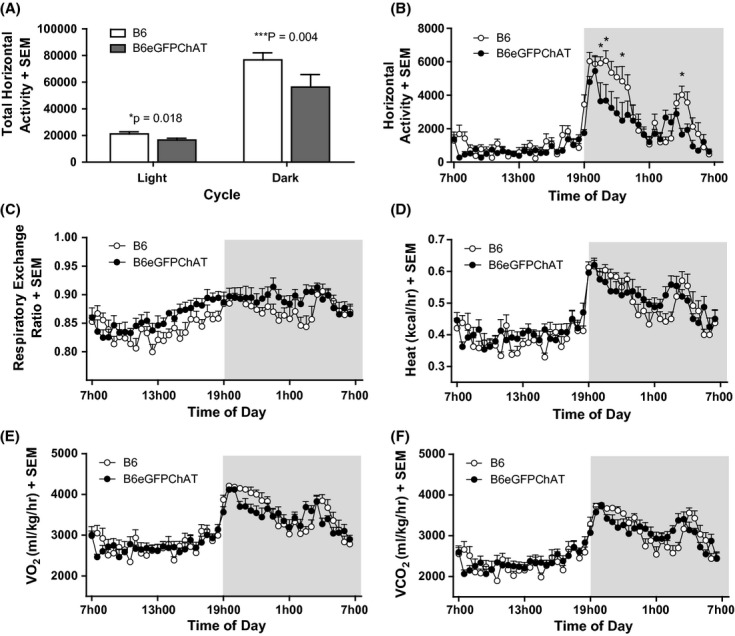
Spontaneous activity and indirect calorimetry of B6eGFPChAT mice. (A) Cumulative horizontal beam break activity over the total each of the 12 h light and dark cycles for B6eGFPChAT (*N* = 8) and B6 control mice (*N* = 8). (B) Home cage horizontal beam break activity recorded in 5 min intervals over 24 h for B6eGFPChAT (*N* = 8) and B6 control mice (*N* = 8). (C) Respiratory exchange ratio recorded in 30 min intervals over 24 h for B6eGFPChAT (*N* = 8) and B6 control mice (*N* = 8). (D) Heat generation recorded in 30 min intervals over 24 h for B6eGFPChAT (*N* = 8) and B6 control mice (*N* = 8). (E) Volume of oxygen (VO_2_) consumed in 30 min intervals over 24 h for B6eGFPChAT (*N* = 8) and B6 control mice (*N* = 8). (F) Volume of carbon dioxide (VCO_2_) generated in 30 min intervals over 24 h for B6eGFPChAT (*N* = 8) and B6 control mice (*N* = 8). Light/dark cycle conditions are distinguished using monochromatic background fill. *P < 0.05; ***P < 0.005 compared with B6 controls.

Given the role of cholinergic neurons in the regulation of muscle activity through central and peripheral innervation, we sought to determine whether increased VAChT expression influences spontaneous locomotor activity. Through the monitoring of locomotor activity over a 24 h period, B6eGFPChAT mice were found to exhibit hypoactivity during both their light (*t*_(14)_ = 2.205; *P* = 0.045) and dark cycles (*t*_(14)_ = 3.823; *P* = 0.002) (Fig. 3A). High-resolution analysis of the locomotor activity exposed a significant genotype factor when analyzed by repeated measures two-way ANOVA (*F*_(1,658)_ = 4.660; *P* = 0.049) (Fig. 3B). Bonferroni post-test revealed that the B6eGFPChAT mice displayed significantly less activity during the biphasic diurnal activity peaks typically exhibited by rodents at ∼2100 and 0430 h (Fig. 3B). We further evaluated physiological function and tone through the assessment of respiratory characteristics that are associated with physical activity. Using two-way repeated measures ANOVA, we found that there was no significant genotype effect during the assessment of RER (*F*_(1,658)_ = 2.105; *P* = 0.169) (Fig. 3C), heat (*F*_(1,658)_ = 0.502; *P* = 0.491) (Fig. 3D), volume of oxygen (VO_2_) (*F*_(1,658)_ = 0.418; *P* = 0.528) (Fig. 3E), and volume of carbon dioxide (VCO_2_) (*F*_(1,658)_ = 0.038; *P* = 0.848) (Fig. 3F). Considering the time points where statistically significant decreases in activity occurred (between 2030 and 2300 h), a corresponding statistically significant decrease in VO_2_ in B6eGFPChAT mice (*F*_(1,70)_ = 5.784; *P* = 0.031) (Fig. 3E) was observed. These results show that B6eGFPChAT mice are spontaneously hypoactive in familiar environments and that locomotion under these conditions is dependent on the expression of VAChT.

### Normal distribution of sleep and wakefulness in B6eGFPChAT mice

**Figure 4:**
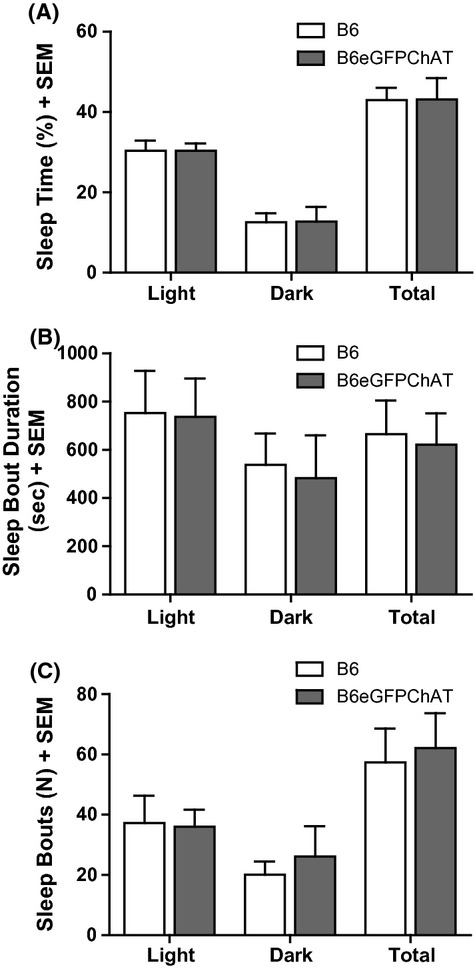
Temporal sleep patterns in B6eGFPChAT mice. (A) Proportion of time spent sleeping during each of the light, dark, and combined cycles over a 24 h period in B6eGFPChAT (*N* = 8) and B6 control mice (*N* = 8). (B) Sleep bout duration during each of the light, dark, and combined cycles over a 24 h period in B6eGFPChAT (*N* = 8) and B6 control mice (*N* = 8). (C) Total number of sleep bouts during each of the light, dark, and combined cycles over a 24 h period in B6eGFPChAT (*N* = 8) and B6 control mice (*N* = 8).

Sleep analysis performed using B6eGFPChAT and B6 control mice home cage activity data did not identify significant genotype factors for the percentage of sleep time (*F*_(1,28)_ = 0.005; *P* = 0.942) (Fig. 4A), average sleep bout duration (*F*_(1,28)_ = 0.389; *P* = 0.538) (Fig. 4B), and the total number of sleeping bouts (*F*_(1,28)_ = 0.771; *P* = 0.387) (Fig. 4C). In addition, the nocturnal preference for wakefulness was maintained between genotypes given the significant cycle factor for sleep time (*F*_(1,28)_ = 363.7; *P* < 0.001), average sleep bout duration (*F*_(1,28)_ = 16.87; *P* < 0.001), and total number of sleep bouts (*F*_(1,28)_ = 24.90; *P* < 0.001). No significant interaction factors were observed for sleep time, duration, or bouts. These data suggest that the duration and circadian patterns of sleep are unaltered by VAChT overexpression in B6eGFPChAT compared with B6 mice.

### B6eGFPChAT mice display increased activity and exhibit impaired habituation in novel environments

**Figure 5:**
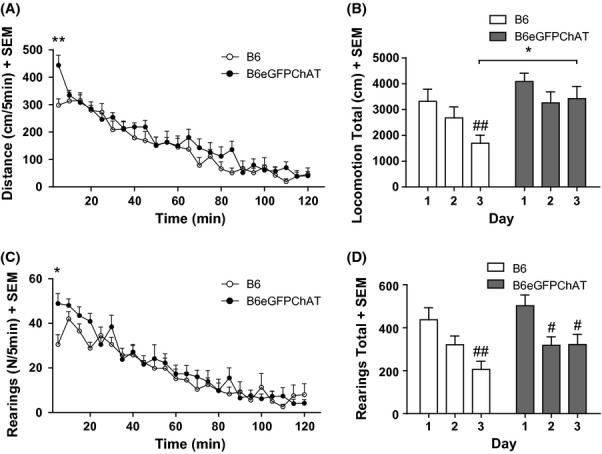
Novel environment locomotion and habituation in B6eGFPChAT mice. (A) Horizontal beam break activity in a novel open field for B6eGFPChAT (*N* = 11) and B6 control mice (*N* = 9). (B) Habituation to the novel open field measured as cumulative 2 h horizontal activity for B6eGFPChAT (*N* = 11) and B6 control mice (*N* = 9). (C) Rearing activity in a novel open field for B6eGFPChAT (*N* = 11) and B6 control mice (*N* = 9). As rearing events are registered, the mouse must go below the level of the vertical sensor for 1 sec before the next rearing event can be recorded. (D) Habituation to the novel open field measured as cumulative 2 h rearing events for B6eGFPChAT (*N* = 11) and B6 control mice (*N* = 9). *P < 0.05; **P < 0.01 compared with B6 controls. ^#^*P* < 0.05; ^##^*P* < 0.01 compared with day 1.

To evaluate the behavioral response to a novel environment, we placed B6eGFPChAT and B6 control mice into open field arenas for 2 h. To establish the instantaneous response to novelty, we first considered the data collected during the initial 5 min of exposure which has been previously established as a predictive time to establish the effect (Crawley 2007). Using this criteria, B6eGFPChAT mice exhibit a significant increase in total distance (*t*_(18)_ = 3.199; *P* = 0.005) (Fig. 5A) and rearing activity (*t*_(18)_ = 2.570; *P* = 0.019) (Fig. 5C) compared with B6 controls.

Activity plots over the 2 h duration of the initial exposure to the novel environment do not reveal significant differences in the intrasession habituation between B6eGFPChAT and B6 controls as expressed by total distance (two-way repeated measures ANOVA revealed significant effect of time, *F*_(23,414)_ = 40.40; *P* < 0.001, but no effect of genotype, *F*_(1,414)_ = 1.210; *P* = 0.286, or interaction, *F*_(23,414)_ = 1.495; *P* = 0.067) (Fig. 5A). Consistently, habituation expressed by rearing events was not statistically different between B6eGFPChAT and B6 control mice (no effect of genotype, *F*_(1,414)_ = 0.445; *P* = 0.513, or interaction, *F*_(23,414)_ = 1.302; *P* = 0.160 and the expected significant effect of time in both strains, *F*_(23,414)_ = 31.53; *P* < 0.001) (Fig. 5C).

In addition to a single exposure, we tested intersession habituation by repeating the exposure of mice to the boxes in three consecutive days (Fig. 5B and D). B6 mice exhibited a decrease in total activity which reached statistical significance during day 3 when compared with day 1 (*F*_(2,26)_ = 5.232; *P* = 0.013) (Fig. 5B; light bars). In contrast, B6eGFPChAT mice did not show statistically significant changes in total distance between exposures (Fig. 5B; dark bars). Notably, B6eGFPChAT mice revealed significantly higher locomotion when compared with B6 control mice during the day 3 exposure (Bonferroni post hoc test between B6eGFPChAT and B6 control on day 3, *t* = 2.884; *P* = 0.013) (Fig. 5B). No significant difference was observed for habituation of rearing events (no genotype effect, *F*_(1,36)_ = 1.405; *P* = 0.251, expected time effect, *F*_(2,36)_ = 17.25; *P* < 0.001) (Fig. 5D). From these data, we show that B6eGFPChAT mice exhibit increased locomotor activity upon initial exposure to open field environments, which decreases to B6 levels by 10 min and is followed by maintained intrasession habituation. In addition, B6eGFPChAT mice were found to have increased locomotor activity compared with B6 controls during the day 3 exposure.

### Thigmotactic behavior is maintained in B6eGFPChAT mice

**Figure 6:**
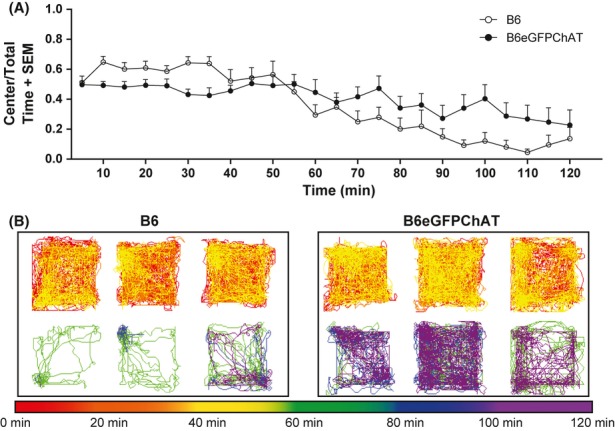
Open field anxiety in B6eGFPChAT mice. (A) Proportion of total time spent in the center of a novel open field environment recorded in 5 min intervals over 2 h for B6eGFPChAT (*N* = 11) and B6 control mice (*N* = 9). (B) Representative cumulative activity plots during the first hour (top row) and second hour (bottom row) for B6eGFPChAT and B6 control mice. Activity is presented in 20 min intervals that have been color coded based on the discontinuous color legend.

We considered that the brief increase in locomotor behavior exhibited in the open field environment might be due to differences in anxiety in B6eGFPChAT compared with B6 mice. We therefore sought to evaluate the thigmotactic behavior of the B6eGFPChAT mice (i.e., the proportion of time spent along the periphery of the open field) during a novel exposure to the environment. No significant difference was observed during the first 5 min (*t*_(18)_ = 0.3479; *P* = 0.732) or during the 2 h duration with regards to the proportion of time spent in the center between the B6eGFPChAT and B6 genotypes (two-way repeated measure ANOVA did not reveal a significant genotype factor, *F*_(1,414)_ = 0.5771; *P* = 0.457) (Fig. 6A). We did observe, however, a significant interaction in the proportion of center time between B6eGFPChAT and B6 control mice (*F*_(1,414)_ = 4.000; *P* < 0.001). Through visual inspection of the data in Figure 6A, we hypothesized that the interaction effect was due to increased unbiased activity during the last hour of the trial. As such, we generated activity maps for the first and second hours of the exposure to compare the activity patterns between genotypes (Fig. 6B). During the first hour of the open field exposure, B6eGFPChAT and B6 genotypes each exhibit unbiased exploration of the open field (Fig. 6B; top row). During the last hour of analysis, B6 mice are found almost exclusively in the peripheral regions of the arena (Fig. 6B; bottom row). In contrast, B6eGFPChAT mice exhibited activity that was unbiased to either the peripheral or center regions of the open field. The pattern of activity and exploration by B6eGFPChAT mice was particularly evident during the last 20 min interval (Fig. 6B; bottom row; purple). These data suggest that enhanced ACh vesicular packaging may contribute to altered thigmotactic behavior through increased activity and exploration to the novel environment.

### B6eGFPChAT mice show increased activity in the dark/light box

**Figure 7:**
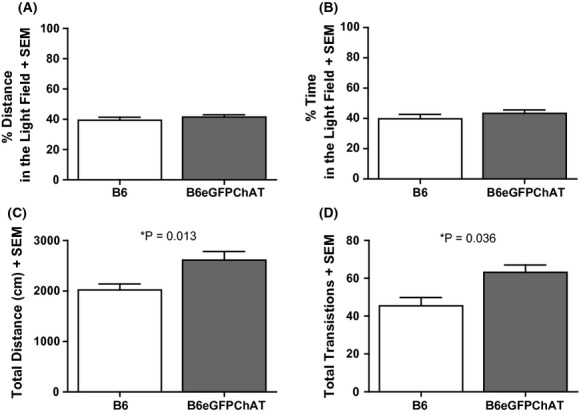
Dark/light box test in B6eGFPChAT mice. (A) Proportion of total distance accumulated in the lit portion of the open field in B6eGFPChAT (*N* = 11) and B6 control mice (*N* = 9). (B) Proportion of total time accumulated in the lit portion of the open field in B6eGFPChAT (*N* = 11) and B6 control mice (*N* = 9). (C) Total distance accumulated in the lit and dark portions of the open field in B6eGFPChAT (*N* = 11) and B6 control mice (*N* = 9). (D) Total transitions between the light and dark portions of the open field in B6eGFPChAT (*N* = 11) and B6 control mice (*N* = 9). *P < 0.05 compared with B6 controls.

To further characterize anxiety levels in B6eGFPChAT compared with B6 mice, the dark/light box paradigm was employed. The dark/light task is based on the innate aversion of mice to brightly lit areas and on the spontaneous exploration of mice in response to mild stressors, in this case novel open environments and light (Crawley 2007). The aversion to the environment is measured by the time and total distance accumulated in each compartment. In this test, B6eGFPChAT and B6 control mice spent ∼40% of their total distance (Fig. 7A) and time (Fig. 7B) in the light compartment and were found not to be significantly different from each other (Mann–Whitney *U* test = 42.00, *P* = 0.649 and Mann–Whitney *U* test = 39.00, *P* = 0.447, respectively). Transitions between the light and dark compartments are considered an index of activity and exploration. In this study, the number of transitions was significantly greater for B6eGFPChAT compared with B6 control mice (Mann–Whitney *U* test = 21.50, *P* = 0.036) (Fig. 7D). Similarly, B6eGFPChAT mice accumulated a significantly greater total distance over the 10 min duration than B6 controls (*t*_(18)_ = 2.740; *P* = 0.013) (Fig. 7C). These results reiterate that B6eGFPChAT mice do not exhibit perturbed anxiety to open environments and light per se, however, B6eGFPChAT mice are more active and display increased exploration to the novel environment of the dark/light box.

### B6eGFPChAT mice exhibit enhanced exploration of the elevated plus maze

**Figure 8:**
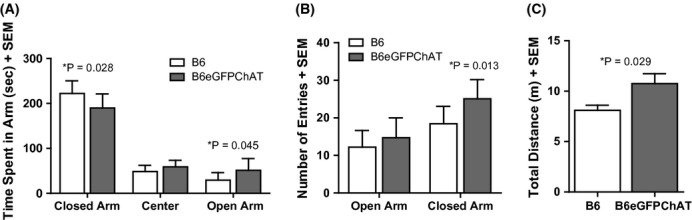
Elevated plus maze performance in B6eGFPChAT mice. (A) Total time spent in the closed, center, and open sections of the elevated plus maze in B6eGFPChAT (*N* = 11) and B6 control mice (*N* = 9). (B) Number of entries into the open and closed arms of the elevated plus maze in B6eGFPChAT (*N* = 11) and B6 control mice (*N* = 9). *P < 0.05 compared with B6 controls.

The elevated plus maze generates an approach/avoidance response using open, elevated arms to measure anxiety-like behavior. Data from the elevated plus maze experiments revealed significant differences in the main parameter indicative of anxiety-like behavior, namely the time spent in the open arm (*t*_(18)_ = 2.150; *P* = 0.045) but not the number of open arm entries (Mann–Whitney *U* test = 36.00; *P* = 0.322) (Fig. 8A and B). Parameters reflecting changes in locomotor activity in this model were also found to be significantly different including the number of closed arm entries (Mann–Whitney *U* test = 16.50; *P* = 0.013) and total distance (*t*_(14)_ = 2.150; *P* = 0.029) (Fig. 8B and C). These data revealed another aspect of the exploratory phenotype to novel environments in B6eGFPChAT mice, as these mice accumulated greater total distance and increased preference to the open arm. The latency to enter the open arm was not used as an outcome measures here as mice were placed into the center of the maze facing one of the open arms.

## Discussion

Here, we present biochemical and behavioral characteristics of B6eGFPChAT mice that delineates the role of VAChT overexpression on cholinergic function, focusing on peripheral motor function, locomotion, and anxiety. Our data provide evidence that modest increases in VAChT expression, previously associated with increased ACh release (Nagy and Aubert 2012), elicits physiological consequences, including spontaneous and novelty-induced locomotor activity. Collectively, these results provide insights on the importance of ACh storage and release on behavior, and this may have implications in human neurodegenerative disorders that exhibit cholinergic dysfunction.

### Biochemical analysis

We previously described that 3-month-old B6eGFPChAT mice have increased VAChT gene and protein expression that results from increased genomic copies of the cholinergic gene locus (Nagy and Aubert 2012). These events are a consequence of the modified RP23-268L19 bacterial artificial chromosome (BAC), containing the VAChT genomic sequence, that was used to initially generate the transgenic mice (Tallini et al. 2006; Nagy and Aubert 2012). Increased VAChT expression enhanced ACh release in the hippocampus (Nagy and Aubert 2012), and likely enhanced cholinergic function in all brain regions where cholinergic terminals are found. Here, we found that VAChT overexpression is maintained at 6 months of age, spanning the age of animals used in this study. In contrast, no significant differences were found for ChAT and CHT protein expression, consistent with our and other's previous findings that alteration in VAChT does not affect other presynaptic cholinergic proteins (Guzman et al. 2011; Nagy and Aubert 2012). VAChT overexpression is therefore maintained at least up to 6 months in B6eGFPChAT mice without affecting ChAT and CHT expression.

### Motor strength and coordination

Spontaneous and evoked release of ACh at the neuromuscular junction is responsible for peripheral muscle contraction in response to motor neuron activation. As such, VAChT-knockdown mice are significantly impaired in their ability to sustain prolonged physical activity, specifically in regards to forelimb grip strength, hanging endurance, and rotarod performance (Prado et al. 2006; de Castro et al. 2009b). An assessment of cholinergic tone at the neuromuscular junction has not been performed in B6eGFPChAT mice. The peripheral expression of the BAC transgene has been previously characterized in B6eGFPChAT mice (Tallini et al. 2006). Using the same mouse model, we found that VAChT is overexpressed in the central nervous system (Fig. 1; Nagy and Aubert 2012) and peripheral regions of the autonomous nervous system (Fig. S1). Our analysis of neuromuscular function in B6eGFPChAT mice reveals that forelimb grip strength and ability to freely support their body weight using an endurance paradigm were maintained. Furthermore, rotarod performance using an accelerating rod to assess coordination, motor learning, and endurance was essentially identical between genotypes. The maintenance of motor function in VAChT-overexpressing mice may reflect the tolerance that exists within the neuromuscular junction to withstand changes in cholinergic transmission. Under normal physiological conditions, peripheral cholinergic neurons maintain cholinergic function through readily releasable pools of ACh-containing synaptic vesicles. During prolonged stimulation, large storage reserves of ACh-containing vesicles can be localized within peripheral cholinergic neurons and used for synaptic release (Rizzoli and Betz 2005). For these reasons, the impact of VAChT overexpression on neuromuscular function may require more demanding physical conditions to be resolved. Indeed, previous studies have identified that CHT overexpression improves performance during endurance treadmill paradigms, while CHT deficiency impaired treadmill performance (Bazalakova et al. 2007; Lund et al. 2010). It remains to be determined whether similar paradigms would elicit an effect in B6eGFPChAT mice.

In contrast to peripheral neurons, central cholinergic neurons have smaller pools of readily releasable vesicles, and as such may be more dependent on the rapid recycling of vesicles. Under certain physiological scenarios, such as when synaptic vesicles cycle faster than they can be filled (Prado et al. 2002), neurotransmitter transporters may be rate limiting to neurotransmitter release. During these events, the rate of ACh release may be enhanced during VAChT overexpression and as such, central cholinergic functions may be more sensitive to modified levels of VAChT.

### Spontaneous activity and circadian rhythms

ACh is known to play a complex role in the regulation of locomotor control, including acting as a modulator of the dopaminergic system (Rice and Cragg 2004; Drenan et al. 2010; Lester et al. 2010; Threlfell et al. 2010). In particular, ACh within the laterodorsal and pedunculopontine tegmental nuclei of the pons has been shown to mediate the dopaminergic activity along the nigrostriatal pathways whose innervation to the dorsal striatum is responsible for voluntary motor control (Lester et al. 2010). In addition, striatal cholinergic interneurons regulate dopamine release via beta2 subunit containing nicotinic acetylcholine receptors (β2*-nAChR) present on dopaminergic axons in the striatum (Threlfell et al. 2010). Several reports show that pharmacological or genetic alteration of cholinergic or dopaminergic function leads to increased striatal dopamine release and increased spontaneous locomotion (Giros et al. 1996; Gomeza et al. 1999; Rice and Cragg 2004; Drenan et al. 2010; Threlfell et al. 2010).

In addition to dopaminergic modulation of locomotion in the striatum, the contribution of forebrain cholinergic tone in spontaneous locomotion has recently been revealed. Mice with VAChT deficiency throughout the central and peripheral nervous system (Martins-Silva et al. 2011) or specifically in basal forebrain neurons (Martyn et al. 2012) display hyperactivity. Interestingly, cholinergic contribution to locomotion appears to be independent of cholinergic striatal interneurons because selective removal of VAChT in the striatum does not induce hyperactivity (Guzman et al. 2011). It is therefore plausible that cholinergic innervation to other central regions, including the cortex and hippocampal formation, play important roles in the regulation of this behavior.

Our findings that B6eGFPChAT mice exhibit hypoactive spontaneous activity are consistent with the notion that ACh “turns down” neuronal circuits controlling spontaneous locomotion (Martins-Silva et al. 2011; Martyn et al. 2012). The observed hypoactivity in B6eGFPChAT mice was most evident during activity peaks occurring over the dark phase of the light/dark cycle. In addition, metabolic parameters of heat, VO_2_, and CO_2_ appear to correspond to daily rhythmic patterns of locomotion, with significant and corresponding decreases in VO_2_ during the periods of significant hypoactivity. The transient decrease in VO_2_ likely reflects the inherent decrease in respiration requirements associated with decreased activity. Taken together, these data suggest that the change in spontaneous activity is closely associated to the activity-rest pattern of B6eGFPChAT mice. These data are consistent with previous findings showing that normal activity-rest patterns are regulated by cholinergic neurotransmission, potentially through β2*-nAChR of the suprachiasmatic nucleus (Liu and Gillette 1996; Yang et al. 2010; Xu et al. 2011). This is because cholinergic neurotransmission is generally associated with a series of characteristic sleep changes, including decreased rapid eye movement sleep (REM) latency and increased REM density (Sarter and Bruno 1999; Vazquez and Baghdoyan 2001). As such, we considered that the sleeping patterns in B6eGFPChAT mice could have contributed to the observed patterns of activity in this study. However, this was found not to be the case, and when activity and inactivity were analyzed by determining movement by infrared beam break (Pack et al. 2007), no significant differences were found in the patterns of sleep time, sleep bout number, or sleep bout duration. Collectively, these data suggest that VAChT overexpression induces generalized locomotor hypoactivity that is unrelated to circadian sleep regulation. VAChT overexpression in B6eGFPChAT mice has not been targeted to specific brain regions, limiting the identification of specific brain areas responsible for the observed hypoactivity. However, based on the discussion above, we postulate that VAChT overexpression is enhancing the inhibitory effect of ACh via cholinergic basal forebrain or dopaminergic striatal networks. Indeed, the decreased spontaneous activity exhibited by B6eGFPChAT mice is reminiscent of mouse models with increased ACh (via AChE inhibition) or decreased dopamine neurotransmission (Kobayashi et al. 1995; Zhou and Palmiter 1995). Confirmation of these potential mechanisms awaits region-specific VAChT overexpression models.

### Exploratory behavior

Novel stimuli, including new or modified environments, generate approach/avoidance conflicts in mice. The conflict tests the balance between exploring the novelty to gain information and the anxiety-related cautiousness to avoid danger or harm. Exposure to novel stimuli has been extensively associated with cholinergic activation. Studies using exposure to novel environments and sensory stimulation as the experimental paradigms have also shown increased ACh release in the nucleus accumbens, hippocampal formation, and cortical structures (Thiel et al. 1998; Schildein et al. 2000; Giovannini et al. 2001). Furthermore, a number of studies have demonstrated that cortical (Day et al. 1991), striatal (Cohen et al. 2012), and hippocampal (Dudar et al. 1979; Day et al. 1991; Mizuno et al. 1991). ACh release is positively correlated to behavioral arousal in novel environments as defined by locomotor activity. We therefore investigated the exploratory behavior in B6eGFPChAT mice in novel environments to evaluate the contribution of VAChT overexpression.

The results from the open field experiments indicate that B6eGFPChAT mice display transient increases in activity upon initial exposure to the novel environment compared to B6 control mice, including increased horizontal activity and rearing. These increased levels of exploration return to normal following the first 10 min of the open field exposure, where B6eGFPChAT mice begin to elicit normal intrasession patterns of habituation. Upon repeated exposure to the novel environment, B6eGFPChAT mice displayed only a modest decrease in locomotion, which did not reach significance, and was found to be significantly different than B6 control mice by day 3. The intrasession and intersession habituation patterns of B6 control mice were found to be consistent with previous reports (Bolivar et al. 2000; Bolivar and Flaherty 2003). While the intrasession habituation of B6eGFPChAT mice was unchanged, the impaired status of *intersession* habituation in this study was unexpected. This is because earlier studies have shown that deficits in habituation are attributed to ACh deficiency (Ukai et al. 1994; Schildein et al. 2000, 2002), as ACh levels in the hippocampus (Giovannini et al. 2001) or cortex (Sarter and Bruno 1999; Sarter and Parikh 2005) may contribute to memory consolidation or attention processes following exposure to the novel environment. We considered that intersession habituation to novel environments may be the result of two components, one related to memory and anxiety and one related to motor activity. Indeed, previous experiments performing repeated exposures to novel environments reveal that during initial exposures, elevated ACh released from cortical and hippocampal regions may be associated with fear, stress, and motor activity (Giovannini et al. 2001). Subsequent habituated exposures have a limited component of memory and anxiety, as the inherent fear elicited by the novelty of the environment is minimized, and as such cortical and hippocampal cholinergic activation is related primarily to motor activity (Giovannini et al. 2001). As such, we propose that the observed intersession activity in B6eGFPChAT mice is attributed to increased exploration associated locomotion of B6eGFPChAT mice exposed to novel environments rather than impaired habituation per se. This speculation can be supported by the observed rearing habituation, which suggests that, to a certain extent, habituation behavior is maintained in B6eGFPChAT. Furthermore, our observed locomotor arousal is consistent with the mechanism that instantaneous release of ACh positively correlates with increased activity in novel environments (Dudar et al. 1979; Day et al. 1991; Mizuno et al. 1991; Cohen et al. 2012), and suggests that VAChT overexpression may potentiate this response.

### Anxiety-like behavior

Endogenous cholinergic tone has been associated with anxiety-like behavior in mice. The effect of ACh is complex in that increased ACh release has been associated with both anxiolytic and anxiogenic actions (File et al. 1998, 2000). For this reason, the relationship between ACh and anxiety may be related to regional subunit configurations of ACh receptors in the central nervous system (File et al. 2000; Labarca et al. 2001; Salas et al. 2003; Gotti and Clementi 2004; McGranahan et al. 2011). In this study, we utilized multiple experimental paradigms (open field, dark/light box, and elevated plus maze) known to elicit behavioral response in mice to assess the role of VAChT overexpression on anxiety-like behavior.

When exposed to a novel open field, B6eGFPChAT mice did not show any center versus peripheral exploratory bias during the first 5 min of analysis, the time that has been previously shown to elicit the most robust anxiety behavior, or over the entire duration of the assay. The strongly significant interaction that was observed during the open field exposure is clarified by considering the activity traces for the test. Whereas each genotype exhibits unbiased exploration of the open field during the first 60 min of analysis, B6eGFPChAT mice show dramatically more exploration in the open field compared with B6 control mice during the final 60 min of analysis. Consistent with these findings, the dark/light box did not differentiate between genotypes with respect to the primary outcomes of time and distance accumulation in the light field. However, an unbiased increase in total distance was revealed for B6eGFPChAT mice that is reflected by an increase in the total transitions between the dark and light fields. Open field and dark/light box did not detect significantly anxiety-like differences between B6eGFPChAT and B6 control mice. However, B6eGFPChAT mice showed a moderate but significant bias to the open arms, suggesting that VAChT overexpression decreased anxiety-like behavior in the elevated plus maze. The decreased anxiety-like behavior observed in the elevated plus maze in the context of the released exploratory inhibition observed during each of the anxiety-like behavioral tasks suggests that the genetic modifications in the B6eGFPChAT have an anxiolytic effect. The divergent findings in the primary outcomes of the open field and dark/light box (no change in anxiety) and the elevated plus maze (decreased anxiety) can be reconciled as the former tasks may not provide the same sensitivity as the elevated plus maze, which delivers a more complex anxiogenic insult (Crawley 2007). Alternatively, changes in the primary outcome of the elevated plus maze during VAChT overexpression may be solely based on the modified exploratory locomotion in the B6eGFPChAT mouse.

### Implications and concluding remarks

In this study, we used congenic B6eGFPChAT mice that are homozygous for the RP23-268L19-EGFP transgene and have been previously characterized as having increased VAChT gene and protein expression (Nagy and Aubert 2012). These commercially available mice have been recently utilized during the investigation of multiple cholinergic pathways primarily for the identification and functional characterization of cholinergic neurons (Ade et al. 2011; Krasteva et al. 2011; Ogura et al. 2011; Rosas-Ballina et al. 2011). Here, we identified that B6eGFPChAT mice present a unique behavioral phenotype compared with B6 controls. While it remains possible that the observed phenotype will be confounded by positional effects related to the random insertion of the BAC transgene, only a single commercially available B6eGFPChAT founder line exists precluding our examination using multiple founders with independent insertion sites. Keeping these limitations in mind, a cholinergic rationale related to the observed increase in VAChT protein and previously defined enhancement in ACh release (Nagy and Aubert 2012) is congruent with the data and it provides a plausible explanation to the observed behavior in B6eGFPChAT mice.

The utility of the B6eGFPChAT mouse as an experimental model for VAChT overexpression could be of significance for future studies related to neurodegeneration. Significant decreases in VAChT expression have been associated with various neurodegenerative conditions (Kuhl et al. 1996; Efange et al. 1997; Bell and Cuello 2006; Bohnen and Albin 2011; Chen et al. 2011). Most notably, progressive VAChT deficiency is observed during AD progression (Bell and Cuello 2006; Chen et al. 2011) and in postmortem AD brains (Efange et al. 1997; Chen et al. 2011). Interestingly, the disease pathology of AD is also marked by abnormal motor behavior including spontaneous hyperactivity and restlessness (Mega et al. 1999; Ognibene et al. 2005; Sterniczuk et al. 2010b; Bedrosian et al. 2011; Walker et al. 2011), as well as enhanced anxiety to novelty (Sterniczuk et al. 2010a; Bedrosian et al. 2011). The series of experiments described in this study suggest that increased VAChT expression observed in B6eGFPChAT mice contributes to spontaneous hypoactive behavior and increased exploration in novel environments. In cases of cholinergic deficiency and impaired locomotor-related behavior, identifying approaches to upregulate VAChT may be of therapeutic significance.

## Supporting Information

**Figure S1.** Vesicular acetylcholine transporter (VAChT) overexpression in the peripheral autonomous nervous system. Whole hearts or the upper one-third of the small intestine were excised from B6eGFPChAT mice and B6 controls, separated by SDS-PAGE, and probed for antibodies targeting VAChT and GAPDH as a loading control. (A) Densitometry (top) of Western blot membranes of whole heart homogenates (bottom) reveal a significant 1.5-fold increase in normalized VAChT immunoreactivity in B6eGFPChAT mice compared to B6 controls. (B) Densitometry (top) of Western blot membranes of small intestine homogenates (bottom) reveal a significant 3.5-fold increase in normalized VAChT immunoreactivity in B6eGFPChAT mice compared to B6 controls. Mean normalized densitometry values were analyzed by Student's *t*-test to compare genotypes. **P* < 0.05 compared to B6 controls.
