# Supplementary material for: B6eGFPChAT mice overexpressing the vesicular acetylcholine transporter exhibit spontaneous hypoactivity and enhanced exploration in novel environments
Source: Brain Behav. 2013 Apr 18;3(4):367–83. doi: 10.1002/brb3.139 (PMC3869679; doi:10.1002/brb3.139)
Supplement: Supplementary file 1 [file brb30003-0367-SD1.docx]

**Supplementary Information**

*Title*: B6eGFPChAT mice overexpressing the vesicular acetylcholine transporter exhibit spontaneous hypoactivity and enhanced exploration in novel environments

*Authors*: Paul Michael Nagy^a,b^, Isabelle Aubert^a,b^

*Affiliations*: ^a^ Brain Sciences Program, Biological Sciences, Sunnybrook Research Institute, 2075 Bayview Avenue, Toronto, Ontario, M4N 3M5, Canada; ^b^ Department of Laboratory Medicine and Pathobiology, University of Toronto, 1 King's College Circle, Toronto, Ontario, M5S 1A8, Canada

**Figure S1**. **Vesicular acetylcholine transporter (VAChT) overexpression in the peripheral autonomous nervous system.** Whole hearts or the upper one-third of the small intestine were excised from B6eGFPChAT mice and B6 controls, separated by SDS-PAGE, and probed for antibodies targeting VAChT and GAPDH as a loading control. **(A)** Densitometry (top) of Western blot membranes of whole heart homogenates (bottom) reveal a significant 1.5-fold increase in normalized VAChT immunoreactivity in B6eGFPChAT mice compared to B6 controls. **(B)** Densitometry (top) of Western blot membranes of small intestine homogenates (bottom) reveal a significant 3.5-fold increase in normalized VAChT immunoreactivity in B6eGFPChAT mice compared to B6 controls. Mean normalized densitometry values were analyzed by Student’s t-test to compare genotypes. * *p*<0.05 compared to B6 controls.
